# Safety assessment of HEA‐enriched *Cordyceps cicadae* mycelia on the central nervous system (CNS), cardiovascular system, and respiratory system in ICR male mice

**DOI:** 10.1002/fsn3.2440

**Published:** 2021-07-16

**Authors:** Hsin‐I Fu, Jui‐Hsia Hsu, Tsung‐Ju Li, Shu‐Hsing Yeh, Chin‐Chu Chen

**Affiliations:** ^1^ Biotech Research Institute Grape King Bio Ltd Taoyuan City Taiwan; ^2^ Institute of Food Science and Technology National Taiwan University Taipei City Taiwan; ^3^ Department of Food Science, Nutrition and Nutraceutical Biotechnology Shih Chien University Taipei City Taiwan

**Keywords:** *Cordyceps cicadae* mycelia, Liquid fermentation, N6‐(2‐hydroxyethyl) adenosine, safety assessment

## Abstract

*Cordyceps cicadae*, an entomopathogenic fungus, is a source of traditional Chinese medicine in China. Due to the low yield of wild *C. cicadae*, artificial cultivation approaches will be needed to meet the increasing market demand. Using bioreactor culture can increase mass production and the abundance of the active component, N6‐(2‐hydroxyethyl)‐adenosine (HEA). Here, we describe a safety assessment for a novel mycelium preparation method. Many studies have confirmed the safety of *C. cicadae* mycelia. However, the acute safety pharmacology of the *C. cicadae* enriched with the high HEA (3.90 mg/g) compound has not been evaluated. This study evaluated the central nervous system (CNS), cardiovascular system, and respiratory system in ICR male mice via oral gavage administration. For each requested item, two batches of eight mice tested on a vehicle (0.5% carboxymethyl cellulose, CMC) and *C. cicadae* mycelia (1,000 mg/kg) were performed. The heart rate at 60 min for the vehicle and *C. cicadae* mycelium treatment was 700.3 ± 55.4 and 603.0 ± 42.3 bpm, respectively (*p* = .4279). For echocardiographic analysis, the LV mass of the vehicle and drug treatment was 86.7 ± 6.4 and 80.2 ± 7.7, respectively (*p* = .0933). In the respiratory test, the tidal volume of the vehicle and drug treatments was 0.11 ± 0.01 and 0.14 ± 0.01 at 60 min, respectively (*p* = .4262). These results demonstrate that the oral administration of HEA‐enriched *C. cicadae* mycelia is safe for the CNS, cardiovascular, and respiratory systems.

## Graphical abstract

Many studies have confirmed the safety of C. cicadae mycelia; however, the acute safety pharmacology of the C. cicadae enriched with the high HEA (3.90 mg/g) compound has not been evaluated. This study evaluated the central nervous system (CNS), cardiovascular system, and respiratory system in ICR male mice via oral gavage administration. The results demonstrate that C. cicadae mycelia have no significant side effect on the cardiovascular, respiratory, and central nervous system via oral administration.

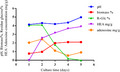

## 1 INTRODUCTION

*Cordyceps cicadae*, also known as the cicadae flower, is a well‐known entomopathogenic fungus. The *C. cicadae* mycelia are parasitic toward specific hosts, such as *Cicada flammata* Distant, *Platypleura kaempferi* Fabricius, *Crytotympana pustulata* Fabricious, *Platylomia pieli* Kato, and *Oncotympana maculatieollis* Motsch (Zeng et al., 2014). The cicadae flower has been used as traditional Chinese medicine (not only in China but also in Japan and Taiwan) for at least 1,600 years to treat fatigue, night perspiration, fever, childish convulsion, palpitation, epilepsy, and many eye diseases (Kuo et al., 2002; Li et al., 2017).

Previous studies have shown that *C. cicadae* can produce many active components, such as adenosine and N6‐(2‐hydroxyethyl)‐adenosine (HEA). HEA is an adenosine derivative; it is associated with control of the brain and coronary circulation and has anti‐inflammatory activity (Deng et al., 2020; Lu et al., 2015). Also, HEA has also been reported to have sedative–hypnotic activity (Chen et al., 2015; Li et al., 2017). The yield of *C. cicadae* collected in the field is low and unstable and easily contaminated with soil mold before drying. Previous studies have shown that the functional composition and physiological activity of artificially cultured liquid‐fermented *C. cicadae* mycelium has a similar effect as wild *C. cicadae* mycelium (Chen et al., 2015; Hsu et al., 2015).

However, liquid fermentation may produce different metabolites due to different cultivation conditions. Thus, a safety evaluation is required. We have previously conducted safety assessments including 90‐day subchronic toxicity (2000 mg/kg bw) (Chen et al., 2015), genotoxicity study, 28‐day subacute toxicity study in LY pig (Jhou et al., 2016), prenatal developmental toxicity study (Li et al., 2017), and acute toxicity study (Lin et al., 2017), and no toxic symptoms have been observed.

In this study, we compare the HEA content from mycelium and various fruiting bodies and investigate the pharmacology safety of HEA‐enriched *C. cicadae mycelia* (1,000 mg/kg bw). To our knowledge, no study has reported a HEA active compound content higher than reported here. This study aimed to investigate the safety of this high HEA content material on the cardiovascular, respiratory, and central nervous systems via oral administration.

## 2 MATERIALS AND METHODS

### 2.1 Materials and chemicals

The *Cordyceps cicadae* sample was collected from the mountainous region of New Taipei City in Taiwan. The sample was cultured on PDA (potato dextrose agar) for 14 days at 25℃ after strain isolation and identification by ITS sequencing (Li et al., 2019). Next, a mycelium agar block (1 cm^3^) was transferred to a 2‐L Erlenmeyer flask with 1.0 L PDB broth and cultured for 3 days on a rotary shaker at 120 rpm and for 25℃. The fermented broth was inoculated into a 500‐L fermenter (BioTop, Taichung, Taiwan) (composed of 1% soybean powder, 5% glucose, and 1% yeast extract) at 25℃, 60 rpm agitation, and 0.5 vvm aeration for 3 days. Then, the broth was scaled up to a 5‐ton fermenter and cultured under the same growth parameters for another 5 days. The fermented HEA‐enriched *C. cicadae* mycelia were harvested, then boiled at 100℃ for 1 hr, freeze‐dried, and ground into a powder. Ultimately, the *C. cicadae* mycelium powder was mixed with the appropriate amount of distilled water before administration to mice.

Following the method described in Chen et al., (2015), the active compound HEA in *C*. *cicadae* mycelia was determined by a high‐performance liquid chromatography equipped with an ultraviolet detector and a reverse‐phase column (Luna 5μ C18(2), 250 × 4.6 mm; Phenomenex, Torrance, CA). The mobile phase contains 10 mmol/L KH_2_PO_4_ and acetonitrile (94:6) with a 1.0 ml/min flow rate. The column was kept at 40℃.

### 2.2 Animals

For each pharmacology safety study, the central nervous system, cardiovascular system, and respiratory system were tested in two batches: Each batch contains a total of 16 mice, with eight in the vehicle control groups and eight in the treatment group. A total of 48 CD1 (ICR) male mice (8–14 weeks old) were obtained and held at the Taiwan Mouse Clinic (Taipei, Taiwan). Different ages of mice were used in the following tests. The mice were allowed free access to a chow diet and reverse osmosis water for accommodation. The facility room environment is kept at 21 ± 2℃, 40%–70% humidity, and a 24‐hr light–dark cycle (LD 12:12). The study protocol was approved by the Institutional Animal Care and Utilization Committee [IACUC number: 13–07–563 (approved on November 19, 2018)]. For the pharmacokinetic (PK) study, 6‐week‐old Sprague Dawley rats were obtained from BioLASCO Taiwan Co., Ltd. After quarantine and accommodation for 1 week, the rats were randomized and were applied in the PK study.

### 2.3 CNS analysis

In brief, the mice were divided into two groups: vehicle control (0.5% CMC) or *C. cicadae* mycelia (1,000 mg/kg). CNS analysis of screening battery contains home‐cage activity (1 hr) (11‐week‐old mice), Modified SHIRPA (11‐week‐old mice), and core body temperature (14‐week‐old mice). All mice were orally administered for 15 min before behavioral testing. Home‐cage activity was recorded for 1 hr, and data were analyzed using Clever Sys HomeCageScan TM3.0. In Modified SHIRPA, the mice were settled in a viewing jar for 5 min. For the core body temperature measurement, the mice's temperature was measure using a rectal probe (KN‐91; Natsume Seisakusho, Tokyo, Japan) before drug treatment (time =0) and 30 and 60 min after administration.

### 2.4 Cardiovascular analyses

In this section, the same dosage concentrations were used as mentioned above, and 8‐week‐old mice were used. Telemetry electrocardiogram (ECG), blood pressure and pulse rate, and echocardiography were included in cardiovascular analyses. Telemetry ECG recording was conducted in their home cage for 15 min before drug treatment; each mouse was then transferred to a novel cage to test their cardiac function after administered via the oral route. Mice implanted with a DSI radiotelemetric transmitter (model TA11ETA‐F10; DSI, St. Paul, MN, USA) were allowed to recover for 1 week. For each drug treatment, an ECG was recorded for 60 min and seven different time points (predrug, 5, 10, 15, 20, 30, and 60 min). The RR interval, heart rate, PR interval, P duration, QRS interval, QT interval, and corrected QT data were collected and analyzed.

For blood pressure analysis, all mice were placed in the channel mouse platform for 4–5 min to allow the temperature to stabilize at 38℃. Mice were then measured for blood pressure by tail‐cuff plethysmography using a BP‐2000 blood pressure analysis system (Visitech Systems Inc., Cary, North Carolina, USA) before drug treatment (time =0), and after drug treatment at 10–15 (15), 25–30 (30), and 55–60 (60) min.

For echocardiography, cardiac structure and function were assessed by high‐frequency echocardiography using a Vevo 3,100 imaging system (VisualSonics, Toronto, Ontario, Canada). Mice were sedated using 2% isoflurane gas with oxygen for 5 min and placed on a prewarmed pad with continuous electrocardiographic monitoring.

### 2.5 Respiratory analyses

The same grouping vehicle control (0.5% CMC) and *C. cicadae* mycelia (1,000 mg/kg) were used for the lung function analysis by mouse whole body plethysmographs (Buxco, Wilmington, North Carolina, USA). Before drug administration, mice were habituated to the Buxco chamber for 15 min; the respiratory activity was recorded for 5 min at the same time as a baseline. Subsequently, we measured the mice's lung functions for 1 hr after drug administration. Ten‐week‐old mice were used in this analysis.

### 2.6 Pharmacokinetic Study

For the pharmacokinetic study, twelve rats were randomly assigned to four groups (*n* = 3) and intravenously administrated with HEA (H4509, Sigma Aldrich, St. Louis, MO, USA) dissolved in PBS buffer (3.0 mg/kg). All animals were anesthetized with Avertin (2,2,2‐tribromoethanol, 0.071 M) before intravenous administration. Blood was collected at 0, 1, 5, 10, 30, 60, 120, 240, 480, and 1,440 min postdose and placed into heparinized test tubes with centrifuge speed at 13,300 × g for 10 min. After blood withdrawal, the brain tissue was collected and homogenized at 0, 30, 60, and 1,440 min postdose. The plasma and brain sample (100 μL) were mixed with 400 μL of methanol. After mixing for 1 min with vortex, the samples were centrifuged at 13,300 × g for 10 min. The supernatant (400 μL) was evaporated to dryness under nitrogen, and the residue was reconstituted with an equal amount of 0.1% formic acid (in DDW). Next, 100 μL of each sample was spiked with 100 μL internal standard solutions (HEA concentration 1 mg/L) for LC‐MS/MS analysis (Marlinge et al., 2017).

### 2.7 Statistical analysis

Continuous data were analyzed by a general linear model, and data ranking was analyzed using a categorical modeling procedure. Fisher's exact test was used for data analysis of Modified SHIRPA, two‐way ANOVA was used for data analysis of core body temperature, blood pressure, lung function, and telemetry ECG, and two‐tailed *t* test was used for data analysis of home‐cage activity and echocardiography. In all cases, p‐values <0.05 were considered significant.

## 3 RESULTS AND DISCUSSION

### 3.1 **Growth curve of liquid‐fermented *C***. ***cicadae***

**FIGURE 1:**
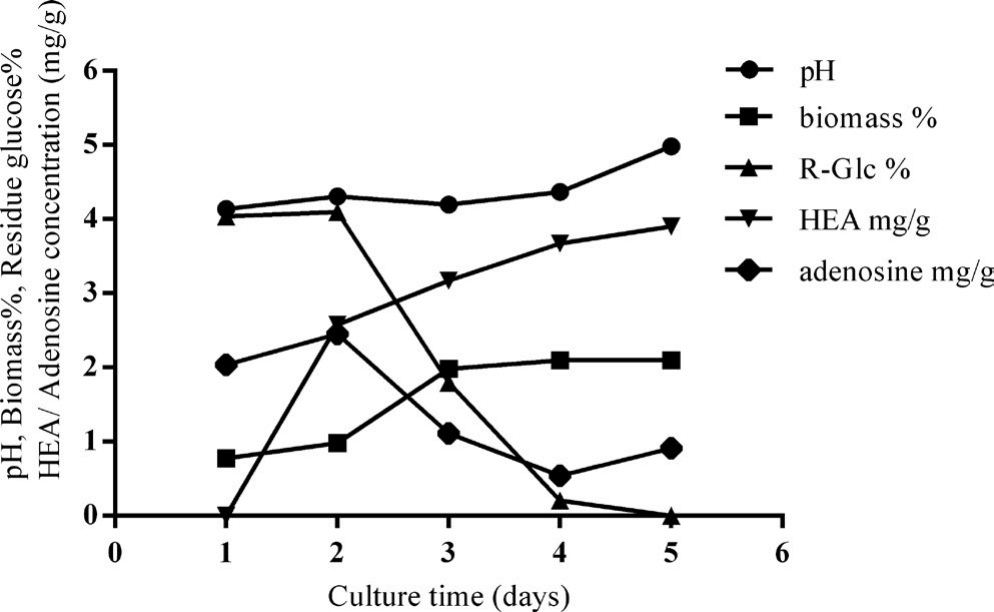
Time course of pH, biomass, residue glucose, HEA, and adenosine contents under 5‐ton liquid fermentation of *C. cicadae*

**FIGURE 2:**
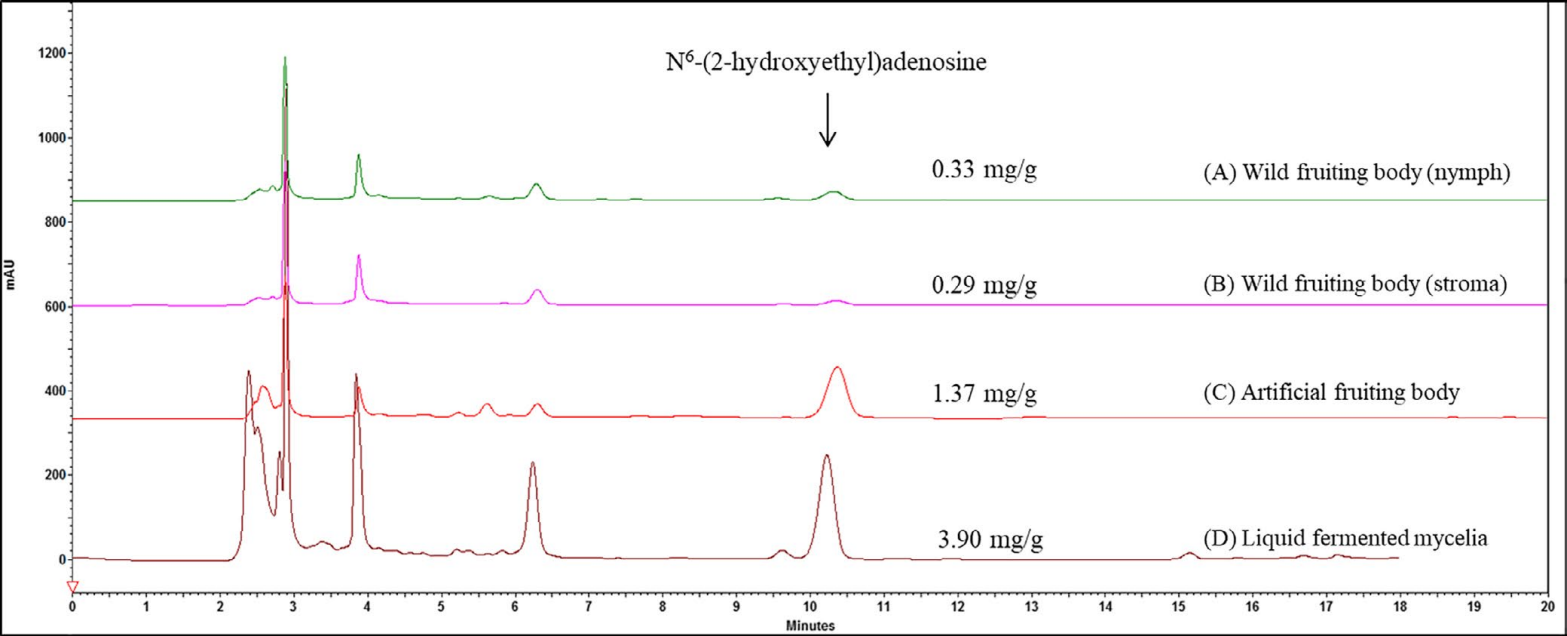
HPLC analysis in *C. cicadae* mycelium and fruiting body. Patterns (a) and (b) were nymph and stroma of wild fruiting body, respectively. (c) The source from the artificial fruiting body and (d) *C. cicadae* mycelium produced from a 5‐ton fermenter

The growth curve of *C. cicadae* cultured in a 5‐ton fermenter is shown in Figure 1. We recorded the growth interval from Day 1 to Day 5. The HEA concentration increased rapidly after Day 1, and the biomass reached the stationary phase at Day 3. After 5 days in culture, 2.10% biomass and 3.90 mg/g HEA content were reached. For residue glucose (R‐Glc) and adenosine, the concentrations start to decrease after Day 2; these two materials were identified as important factors for biomass growth and metabolism (Ke & Lee, 2019). We observed that the adenosine concentration decreased during the fermentation; this phenomenon may be due to the conversion of HEA because HEA has been reported as an adenosine analog (Meng et al., 2015). HPLC was used to analyze the HEA concentration of liquid‐fermented mycelia and artificial and wild fruiting bodies (Figure 2). The HEA content of liquid‐fermented *C. cicadae* mycelium was 3.90 mg/g, and that in wild and artificial fruiting bodies was 0.29 and 1.37 mg/g, respectively. The values for the other group's fruiting body and liquid fermentation were 1.10 and 1.36 mg/g, respectively (Ke & Lee, 2019). Compared with artificial and wild *C. cicadae* fruiting bodies, liquid‐fermented *C. cicadae* mycelium possesses the highest HEA value, and no research report has been found with a higher content than this.

### 3.2 Safety assessment

According to Zhao et al., (2020), a test article in safety pharmacology should be used at or above the therapeutic dose to investigate the potential adverse effect. With regard to safety pharmacology studies, the most important evaluation includes central nervous systems, cardiovascular and respiratory, which functions with respect to life‐supporting functions (Abraham, 2010). The dosage in this study (*C. cicadae mycelium* 1,000 mg/kg ICR mice) that converts to a normal adult human having a body weight of 70 kg is 5.69 g per day, higher than the previous study dose (1.05 g/day/person) (Tsai et al., 2020). To our knowledge, no study has reported the pharmacology safety profile of this high amount of HEA. To address this, in this study, we have investigated the safety assessment of *C. cicadae* mycelia on the central nervous, cardiovascular, and respiratory systems.

### 3.3 CNS analyses

**FIGURE 3:**
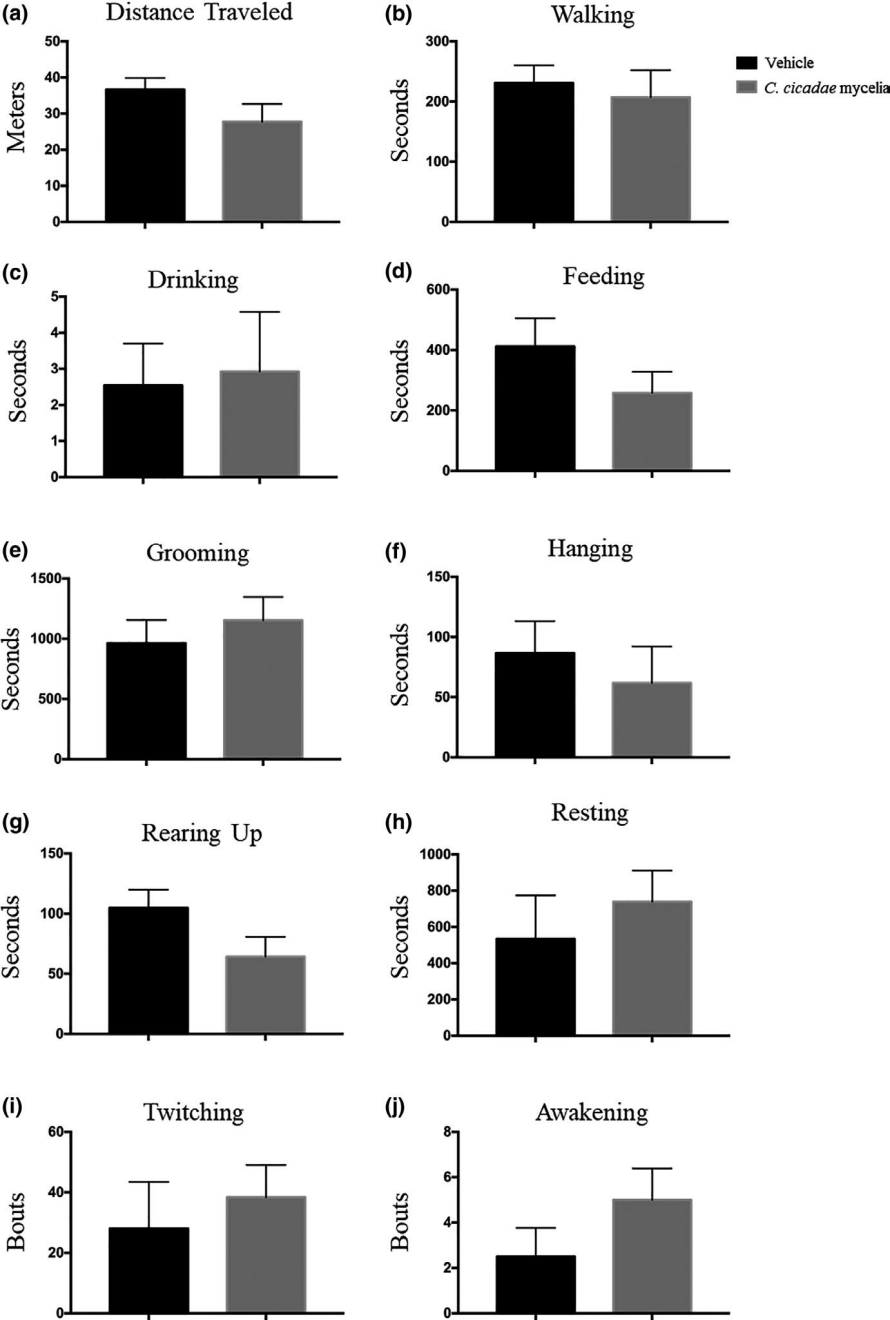
Effects of vehicle (0.5% CMC) and *C. cicadae* mycelia (1,000 mg/kg) on home‐cage activity in ICR mice, (a) including distance traveled, (b) walking, (c) drinking, (d) feeding, (e) grooming, (f) hanging, (g) rearing up, (h) resting, (i) twitching, and (j) awakening. Data are presented as mean ± *SEM* (*n* = 8)

For home‐cage activity analysis, a fully automatic analysis system was used to study unconstrained mouse behaviors, which included distance traveled, walking, drinking, feeding, grooming, hanging, rearing up, resting, twitching, and awakening. There were no significant differences between the two groups in all ten home‐cage behaviors (Figure 3). Moreover, we notice a trend in the *C. cicadae* mycelium group with lower food consumption, which may be due to their food consumption. *C. cicadae* mycelia have abundant fiber. Hanging and rearing up behaviors are related to intensity activity strength (Luby et al., 2012). The *C. cicadae* mycelium group has a trend with lower hanging and rearing up behaviors, but the results between test groups showed no statistical difference. Next, modified SHIRPA is a three‐stage protocol that was used to detect a wide range of behavioral, neurological, and physiological measures in the same group of animals (Zhang et al., 2016). In this study, it provides a preliminary analysis of the morphological and behavioral characteristics of mice. A total of 43 behavioral assessments of mice were recorded (Table 1). No significant differences were found in all behavior items between vehicle and *C. cicadae* mycelium group by Fisher's exact test (*p* = 1.0000).

**TABLE 1:** Modified SHIRPA in vehicle and *C. cicadae* mycelium treatment

Category	Tests	Vehicle (*n* = 8)	*C. cicadae* mycelia (*n* = 8)
Appearance	Skin color	*N*	*N*
Posture	body position; body tone; abdominal tone; visual placing; tail and pelvic elevation; trunk curl	*N*	*N*
Activity	spontaneous and locomotor activity; negative geotaxis; gait	*N*	*N*
Behavior	Fear; Irritability; Aggression; Vocalization; Bizarre behavior; Convulsions; tremor	*N*	*N*
Reflexes	startle response; limb grasping; grip strength; positional passivity; corneal reflex; righting reflex; touch escape; transfer arousal	*N*	*N*
Body releases	defecation; urination; lacrimation; salivation	*N*	*N*
Heart rate		*N*	*N*
Respiration rate		*N*	*N*
Body weight (g)		37.5 ± 1.0	39.3 ± 0.6

Abbreviation: *N*, normal.

**FIGURE 4:**
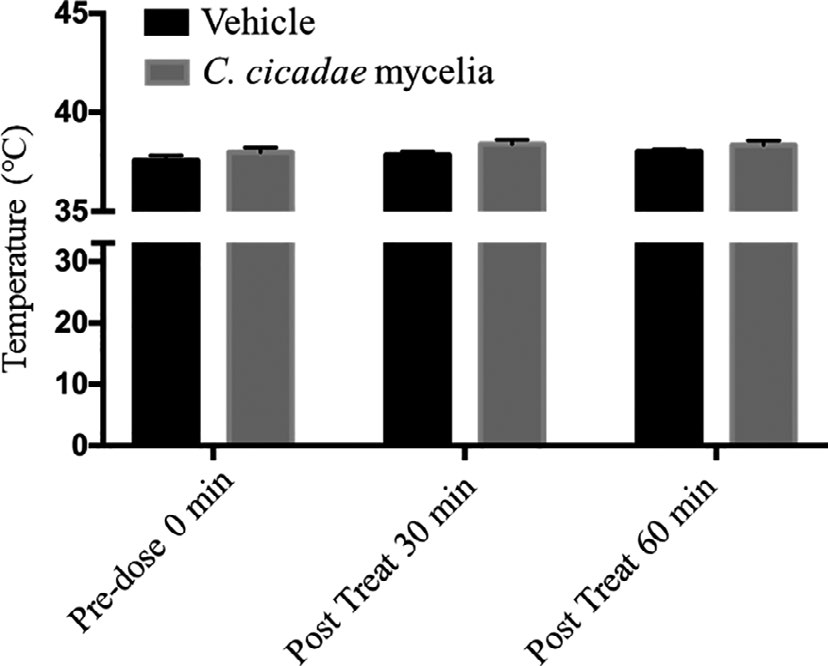
Effects of core body temperature in ICR mice. Data are presented as mean ± *SEM*. versus. vehicle (0.5% CMC, *n* = 8; *C. cicadae* mycelia 1,000 mg/kg, *n* = 8)

For the core body temperature in ICR mice, the rectal temperatures at time 0 in the vehicle group and the *C. cicadae* mycelium group were 37.6℃ and 38.0℃, respectively. At 30 and 60 min, after the treatment had been administered, the body temperatures of the vehicle were 37.8℃ and 38.0℃, and of *C. cicadae* mycelium treatment were 38.4℃ and 38.3℃, respectively (Figure 4). Based on a normal distribution of temperatures measured using a rectal probe (Fiebig et al., 2018; Saegusa & Tab ata, 2003), we found no significant interaction effect and no significant treatment differences between the two groups by using two‐way ANOVA analysis.

### 3.4 Cardiovascular analyses

In this section, some cardiovascular function‐related indicators, including telemetry ECG, blood pressure, and echocardiography, were investigated.

#### 3.4.1 Telemetry ECG

**FIGURE 5:**
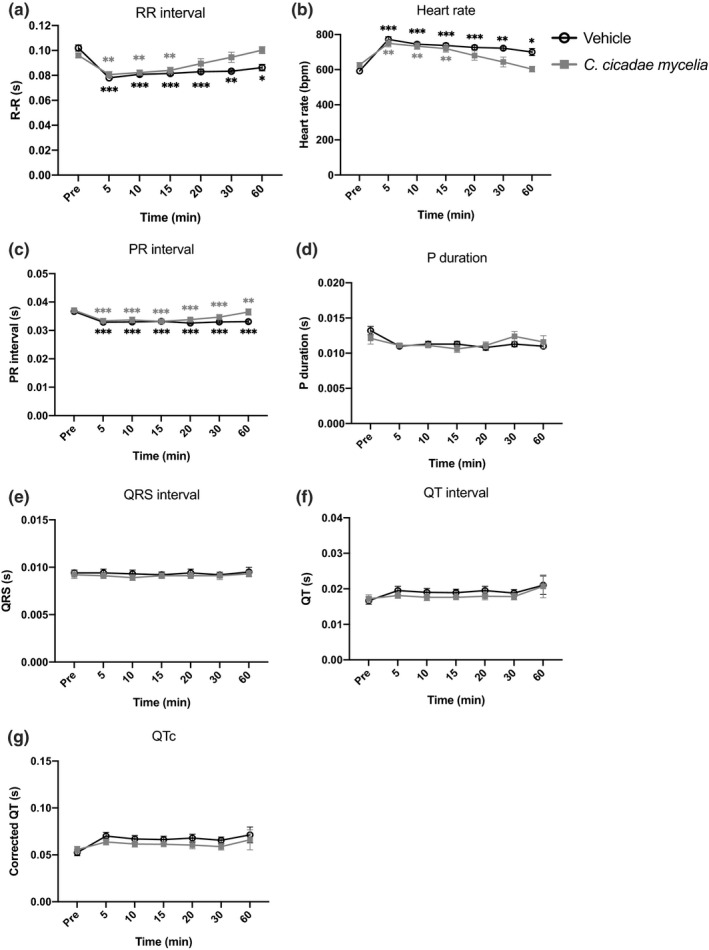
Effects of telemetry ECG in ICR mice. ECG recording of (a) RR interval, (b) heart rate, (c) PR interval, (d) P duration, (e) QRS interval, (f) QT interval, and (g) corrected QT. Statistically, repeated‐measures two‐way ANOVA was used to compare between groups. The post‐Bonferroni analysis presented in the figure of A, B and C, * and *p <.05, ** and **p <.01, and *** and ***p <.001 compared with basal level of predrug. Data are presented as mean ± *SEM*. (vehicle, *n* = 8; *C. cicadae* mycelia 1,000 mg/kg, *n* = 8)

Normally, freely roaming mice have a heart rate (HR) of 550–725 beats per min (bpm) and a maximal HR of 725–815 bmp. In mice, the PR interval amounts to 30–56 ms, the QRS complex has a duration of 9–30 ms, and the corrected QT interval (QTc) ranges between 30 and 124 ms (Kaese et al., 2013). In our study, ECG recording was tested in two different groups, vehicle and *C. cicadae* mycelium treatment. The results showed that *C. cicadae* mycelium treatment had no obvious adverse effect on ECG. There were no significant changes observed in P duration (*p* =.2215), QRS interval (*p* =.8571), QT interval (*p* =.9536), and corrected QT (*p* =.7818) between both treatment groups (Figure 5). Of note, in the telemetry ECG assays, mice showed a temporal increase in heart rate (and decrease in RR interval) due to a response to handling. Since each mouse was transferred to a novel cage to test their cardiac function after administered, this may explain a temporal rapid heart rate due to this transfer event. *C. cicadae* mycelium‐treated mice demonstrated a fast recovery to their basal state levels on RR interval (*p* <.0001), heart rate (*p* <.0001), and PR interval (*p* =.0082) when compared to its vehicle after 20 min. This fast recovery could also come from the *C. cicadae* mycelia's natural compound, such as HEA (N6‐(2‐hydroxyethyl)‐adenosine). HEA is a Ca^2+^ antagonist; it may help to reduce the heart rate and depress contractility under physiological conditions in cardiovascular disorders (Furuya et al., 1983; Hsu et al., 2015). However, since this is a one dosage experiment, further long‐term consumption will be needed to verify this phenomenon. Together, these data suggest *C. cicadae* mycelia has almost no effect on cardiac function.

#### 3.4.2 Blood pressure

**FIGURE 6:**
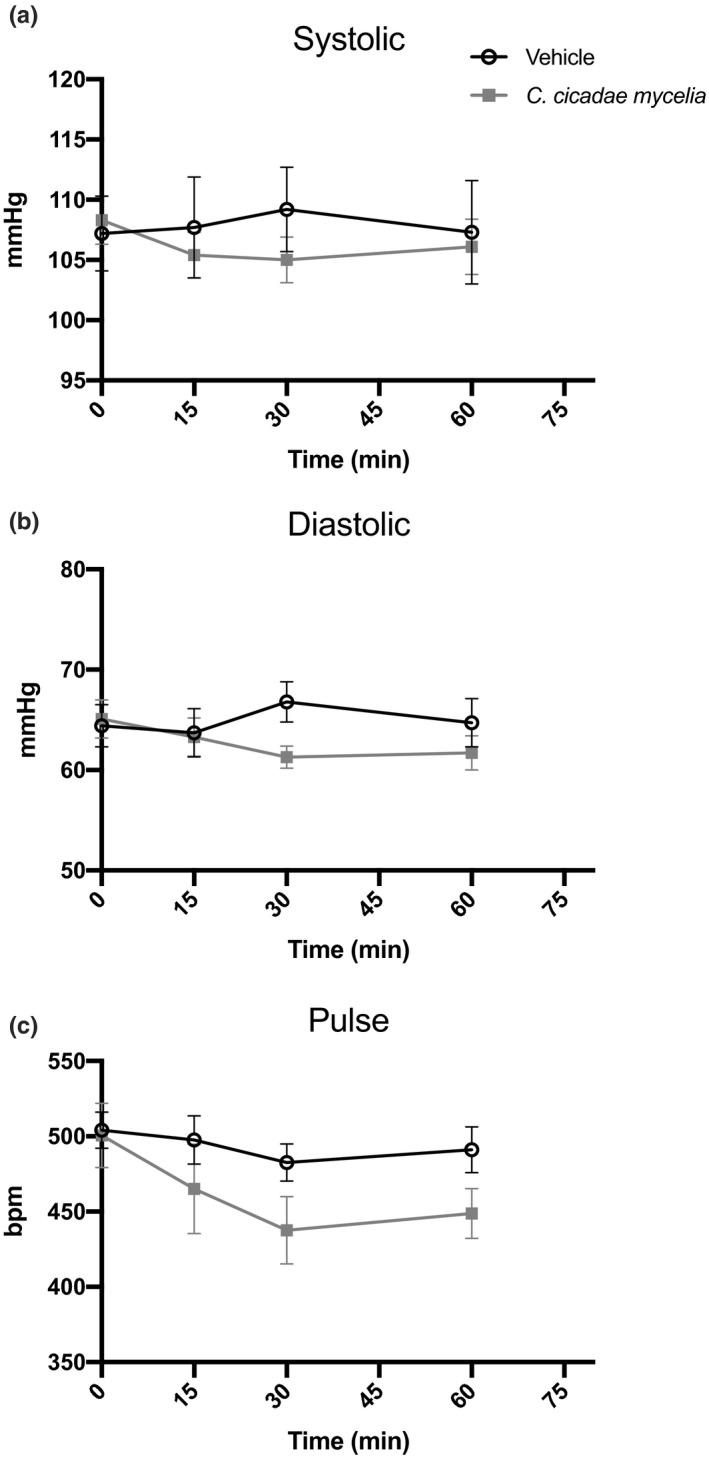
Systolic (a) and diastolic (b) blood pressure and heart rate (c) in vehicle‐ and *C. cicadae* mycelium‐treated mice were measured by a tail‐cuff method. Data are mean ± *SEM* of eight mice (five recording sessions each) per group. bpm indicates beats per min

For blood pressure, systolic and diastolic pressure and pulse were investigated. These parameters fluctuate within the normal physiological range. Compared with vehicle, the *C. cicadae* mycelium group was not significantly different in the systolic (*p* =.6422) and diastolic (*p* =.1283) blood pressure or pulse rate (*p* =.4279) responses (Figure 6). The *C. cicadae* mycelium group had a slightly lower trend within the systolic and diastolic values and pulse rates. Although no significant difference was found, this decreasing trend may be due to the HEA contents of the *C. cicadae* mycelium treatment. According to Furuya et al. (1983), HEA has been reported as a nature calcium antagonist and inotropic agent. And it has been reported that calcium antagonist can reduce cytosolic free calcium concentration vasodilation in the coronary beds and has lower blood pressure effects (Grossman & Messerli, 2004).

#### 3.4.3 Echocardiography

**FIGURE 7:**
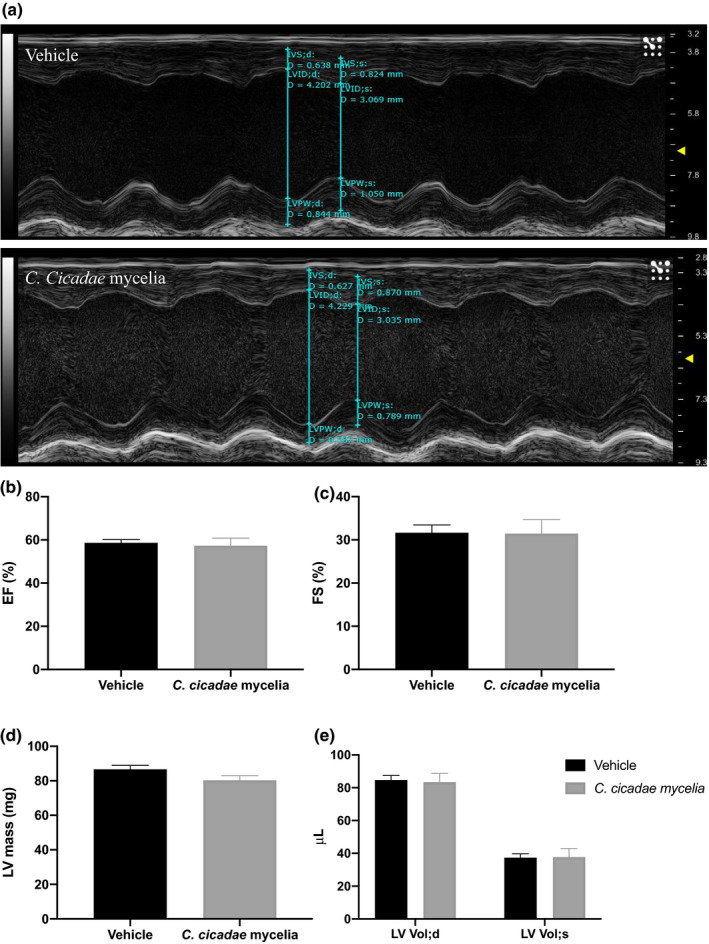
Transthoracic echocardiography evaluating the left ventricular structure and function of mice following vehicle and *C. cicadae* mycelium treatments. (a) Representative M‐mode recordings of echocardiography. (b‐e) Quantitative analysis of the ejection fraction (EF), fraction shortening (FS), left ventricular mass (LV mass), and the diastolic and systolic left ventricular volume (LV Vol; d and LV Vol; s) of ICR male mice by echocardiography following vehicle and *C. cicadae* mycelium treatments. Values are means ± *SEM*

Echocardiography has been widely applied in determining cardiac functions and phenotypes in murine models. The technique is convenient and safe, and allows consecutive and repeated evaluation of cardiovascular physiological and pathologic characteristics in live animals (Wu et al., 2010). Global and regional left ventricular (LV) functions are well‐known indicators of cardiac disease (Salm et al., 2006). The most commonly used indexes of global LV systolic function are fractional shortening (FS) and ejection fraction (EF) because they are easily measured (Chengode, 2016; Liu & Rigel, 2009). FS and EF were positively correlated, and the higher value means a better heart contraction (Child et al., 1981; Yoshikawa et al., 2012). LV mass is the weight of the left ventricle, which is a commonly used descriptor of cardiac status (Pickering, 2007). For the echocardiography, the left ventricular (LV) structure and function of mice following vehicle and *C. cicadae* mycelium treatments were evaluated by transthoracic echocardiography. The echocardiographic data of two treatments are shown in Figure 7a; the left ventricular (LV) function remains the same between the vehicle and *C. cicadae* mycelium group. The results of ejection fraction (EF), fraction shortening (FS), left ventricular mass (LV mass), and the diastolic and systolic left ventricular volume of ICR mice by echocardiography following vehicle and *C. cicadae* mycelium treatments showed no significant changes (Figure 7b‐e).

### 3.5 Respiratory analyses

**FIGURE 8:**
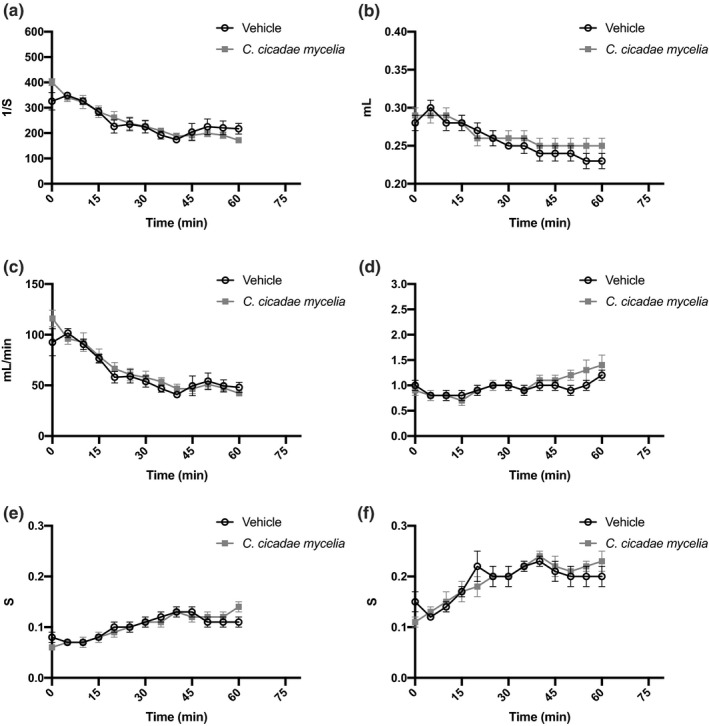
Effects of *C. cicadae* mycelia on lung function in ICR mice. The following parameters were assessed: (a) frequency (f), (b) tidal volume (TVb), (c) minute volume (MVb), (d) respiration resistance (Penh), (e) inspiratory time (Ti), and (f) expiratory time (Te). Data are presented as mean ± *SEM* (vehicle, *n* = 8; *C. cicadae* mycelia, *n* = 8)

Respiration is a vital function. Therefore, it is required to be assessed in the safety analysis. This is to check whether the test substance might affect airway function (Goineau et al., 2013). In this study, the function examines the lung using noninvasive handling to evaluate which examines volume change in particular space (chamber) to count the alteration of respiration gas volume of a mouse. The results show no significant differences between vehicle‐ and *C. cicadae* mycelium‐treated mice in all lung function parameters, including breath frequency (f) (*p* =.3044), tidal volume (TVb) (*p* =.4262), minute volume (MVb) (*p* =.5215), respiration resistance (Penh) (*p* =.2199), inspiratory time (Ti) (*p* =.3096), and expiratory time (Te) (*p* =.5874) between vehicle‐ and *C. cicadae* mycelium‐treated groups (Figure 8). Furthermore, we observed a trend in the *C. cicadae* mycelium group with higher tidal volume (TVb) and respiration resistance (Penh). This might be due to *C. cicadae* mycelium HEA contents, which appears to have a sedative function in pharmacological tests (Liu et al., 2017; Wang et al., 2013). According to Hitomi et al., (2019), an increase in TVb can have a positive impact on health, such as subjective stress‐related feelings and regulating the autonomic nervous system.

### 3.6 Pharmacokinetic study

**FIGURE 9:**
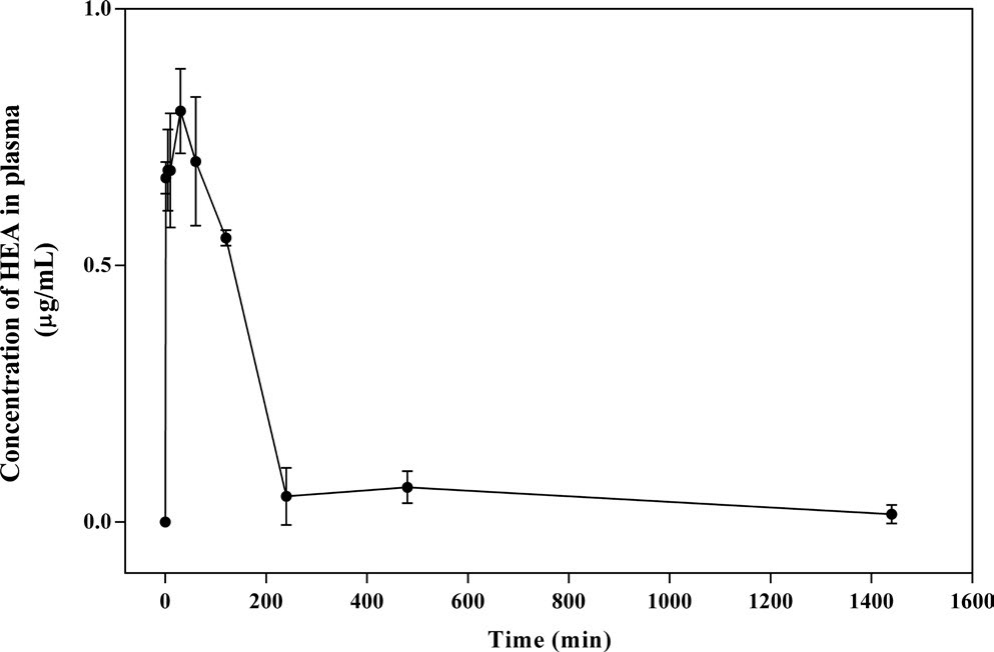
Plasma concentration–time curves of HEA in rats after intravenous administration of HEA at 3.0 mg/kg. Values are means ± *SD* (*n* = 3)

**FIGURE 10:**
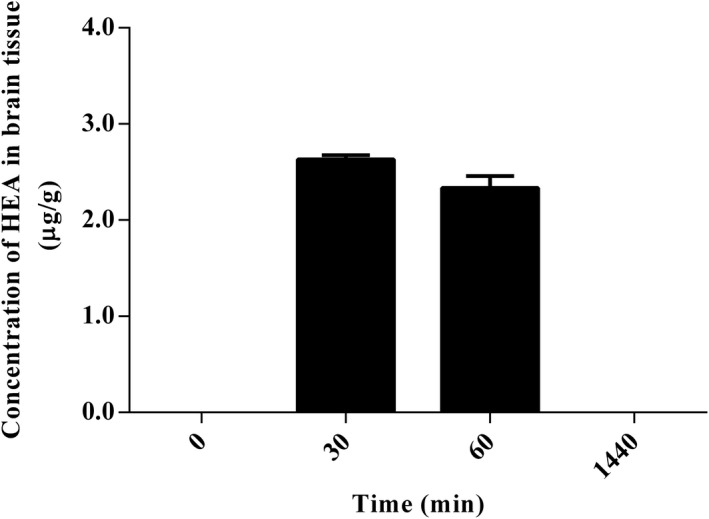
Brain biodistribution at time 0, 30, 60, and 1,440 min of HEA in rats after intravenous administration of HEA at 3.0 mg/kg. Values are means ± *SD* (*n* = 3)

To evaluate the test time used in this study, we conducted a pharmacokinetic study of intravenous injection of the HEA. The developed LC‐MS/MS method was successfully applied to monitor the time course of HEA (3.0 mg/kg). The plasma concentration–time profiles after intravenous administration are shown in Figure 9. After intravenous administrations, the maximum plasma concentration of HEA was 0.80 ± 0.08 μg/mL at 30 min and began to drop afterward. As for brain biodistribution profiles after intravenous administrations (Figure 10), the maximum concentrations of HEA were 2.63 ± 0.04 and 2.34 ± 0.12 μg/g at 30 and 60 min, respectively. After 1,440 min (24 hr), HEA concentrations in the brain of rats were undetectable. This phenomenon shows that HEA is retained in the brain for at least over 60 min, and it can be metabolized completely after 1,440 min. The dose of HEA by intravenous injection shows no toxicity signs were observed in rats during this pharmacology test.

## 4 CONCLUSION

Based on the comprehensive safety pharmacology analysis, no significant adverse effects of *C. cicadae* mycelia were observed compared with the vehicle. In the telemetry ECG section, compared with the vehicle, *C. cicadae* mycelium‐treated mice demonstrated a fast recovery to their basal state levels on RR interval, heart rate, and PR interval, suggesting that *C. cicadae* could benefit those with cardiovascular disorders. In conclusion, we believe *C*. *cicadae* mycelia is safe to consume at a high dose level (1,000 mg/kg) with HEA at 3.9 mg/g for a future functional nutrient ingredient, and this value is higher than the current US New Dietary Ingredient (Bio‐Cordyceps GK‐4 NDI No. 834). In future work, we will process this research and scale up to 20 tons and analyze its HEA composition to see whether we can improve this value further.

## CONFLICT OF INTEREST

The authors declare that they do not have any conflict of interest.

## AUTHOR CONTRIBUTIONS

Conceptualization, C.‐C.C. and S.‐H.Y.; Data curation, H.‐I.F. and T.‐J.L.; Methodology, J.‐H.H.; Resources, J.‐H.H.; Supervision, C.‐C.C.; Writing–original draft, H.‐I.F.; Writing–review & editing, H.‐I.F. and T.‐J.L. and C.‐C.C. All authors have read and agreed to the published version of the manuscript.

## ETHICAL APPROVAL

The study was carried out after the study's protocols and approved from the Institutional Animal Care and Utilization Committee [IACUC number: 13–07–563 (approved on November 19, 2018)].

## Data Availability

The data that support the findings of this study are available on request from the corresponding author.
